# Open laminectomy vs. minimally invasive laminectomy for lumbar spinal stenosis: a review

**DOI:** 10.3389/fsurg.2024.1357897

**Published:** 2024-11-07

**Authors:** Utpal K. Dhar, Emma Lilly Menzer, Maohua Lin, Timothy O’Connor, Nischal Ghimire, Elias Dakwar, Ioannis D. Papanastassiou, Kamran Aghayev, Chi-Tay Tsai, Frank D. Vrionis

**Affiliations:** ^1^ Department of Ocean and Mechanical Engineering, Florida Atlantic University, Boca Raton, FL, United States; ^2^ Department of Neurosurgery, Marcus Neuroscience Institute, Boca Raton Regional Hospital, Boca Raton, FL, United States; ^3^ Department of Orthopedic, Tribhuvan University Teaching Hospital, Kathmandu, Nepal; ^4^ Department of Orthopedic, General Oncological Hospital Kifisias “Agioi Anargryroi”, Athens, Greece; ^5^ Department of Neurosurgery, Biruni University, Istanbul, Turkey

**Keywords:** laminectomy, minimum invasive surgery, lumbar spinal stenosis, finite element analysis, human lumbar spine

## Abstract

**Objectives:**

Lumbar spinal stenosis (LSS) refers to a narrowing of the space within the spinal canal, which can occur at any level but is most common in the lumbar spine. Open laminectomy and minimally invasive laminectomy (MIL) procedures are the most common surgical gold standard techniques for treating LSS. This study aims to review clinical and biomechanical literature to draw comparisons between open laminectomy and various MIL techniques. The MIL variation comprises microendoscopic decompression laminotomy, unilateral partial hemilaminectomy, and microendoscopic laminectomy.

**Methods:**

A review was performed according to the Preferred Reporting Items for Systematic Reviews and Meta-Analyses guidelines. We reviewed 25 clinical, 6 finite element, and 3 cadaveric studies associated with treating LSS. We reviewed literature that discusses factors such as operation time, length of hospital stay, postoperative complications, reoperation rate, effect on elderly patients, patients’ satisfaction, and adjacent segment disease degeneration for the clinical studies, whereas the range of motion (ROM), von Mises stresses, and stability was compared in biomechanical studies.

**Results:**

MIL involves less bone and ligament removal, resulting in shorter hospital stays and lower reoperation and complication rates than open laminectomy. It improves the quality of health-related living standards and reduces postoperative pain. Biomechanical studies suggest that laminectomy and facetectomy increase annulus stress and ROM, leading to segmental instability.

**Conclusion:**

Although theoretically, MIL means less tissue injury, pain, and faster recovery in the short term, the long-term results depend on the adequacy of the decompression procedure and tend to be independent of MIL or open laminectomy.

## 1 Introduction

Lumbar spinal stenosis (LSS) is a consequence of degeneration of the lumbar spine, categorized by narrowing of the spinal canal and the neural foramina. This narrowing can cause compression of the spinal cord and nerve roots running through these channels resulting in potentially severe pain, motor, and sensory deficits (1, 2). LSS is a common condition primarily affecting older adults due to age-related spine degeneration (1, 3). Stenosis is more likely to affect the lumbar spine than other regions, and research suggests that the probability of stenosis occurring in the lumbar spine is about five times that of the cervical spine (4). Generally, mild stenosis occurs in 22.6%–77.9% of people whereas moderate or severe stenosis takes place in 8.40%–30.4% of the population over the age of 40 (2). Open surgical laminectomy and minimally invasive decompression (MID) surgery are two common procedures for treating LSS in the absence of instability (5–11).

LSS can have substantial implications for patients and is known to be a significant driver of morbidity in the older population (12–15). There is considerable overlap in the main surgical approaches for these conditions, two of which are minimally invasive laminectomy (MIL) techniques and open laminectomy (8, 10, 16–19). MIL has become increasingly popular in recent decades due to its cost-effectiveness (20). Additionally, some research suggests that MIL techniques may also provide benefits such as reduced blood loss, shortened hospital stays, reduced pain, and improved quality of life (7, 10, 21, 22). Laminectomies, facetectomies, and MIL techniques are common methods that aim to decompress the lumbar spine in cases of LSS. Historically, open laminectomies have been the standard treatment for LSS (23–27). This procedure attempts to relieve symptoms by removing various structures, including the spinous process and laminae; however, several limitations and disadvantages are associated with this approach. For example, some clinical research has demonstrated postoperative instability, degenerative disease, and lumbar spine deformity, among other potential complications (22, 28–30). On the other hand, MIL techniques aim to minimize tissue disruption and preserve most of the posterior elements to potentially provide better load-bearing capacities and biomechanical stability (31–34).

In addition to clinical research, biomechanical research and stability analyses also provide critical insights. Various *in vitro* studies using human cadavers have been conducted to explore lumbar spine stability. However, this approach has limitations, such as anatomical differences between cadavers (35). Finite element analyses (FEAs) can overcome various limitations given their efficiency, consistency, and reproducibility. FEAs are a powerful tool for studying different segment motions (rotational or translational) and stress–strain relationships and providing necessary parameters for a fuller understanding (18, 31, 36). FEAs offer distinct insights from a biomechanical standpoint and can provide surgeons with valuable insights to facilitate future surgical procedures (33, 37–41). Few review papers were published in clinical studies that correlate with the biomechanical studies for the cervical spine (42, 43). The present study aims to analyze and compare the literature between laminectomy and minimally invasive surgery for both clinical and biomechanical aspects of human LSS.

## 2 Methods

### 2.1 Search strategy

**Figure 1:**
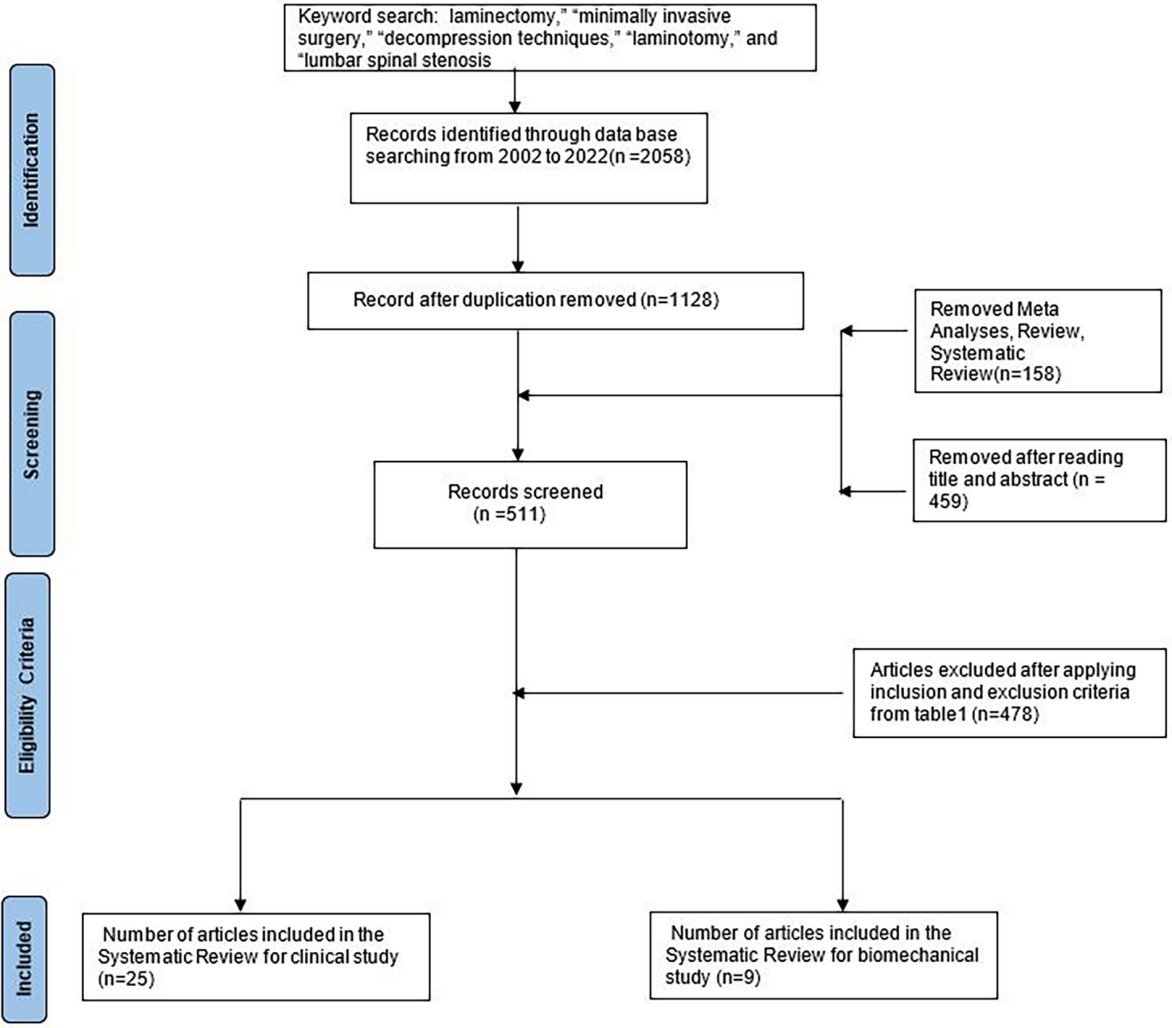
Search strategy for our review.

Our review followed the PRISMA instruction for selecting the literature (44). A search was conducted in PubMed for articles containing the keywords “laminectomy,” “minimally invasive surgery,” “decompression techniques,” “laminotomy,” and “lumbar spinal stenosis.” A flow diagram of how the clinical and biomechanical studies were selected for the review is shown in Figure 1.

### 2.2 Screening

All titles and abstracts were first examined, and those that did not meet the criteria in Table 1 were removed. Subsequently, all remaining articles were reviewed in full. Studies were limited to only those looking at the human lumbar spine and written in the English language. Studies of the thoracic and cervical spine were also excluded. In the current study, MIL variance included such as microdecompressive laminotomy (MDL), windows technique (WT), unilateral microendoscopic laminectomy (MEL), microendoscopic posterior decompression (MEPD), minimally invasive decompression, unilateral partial hemilaminectomy (UPHL), and spinous process splitting decompression (SPSD). The MIL techniques that involved fusion with internal instrumentation and interbody cages were excluded in this review.

**Table 1:** Exclusion and inclusion criteria for selecting literature.

Inclusion criteria	Exclusion criteria
The article focused on human lumbar spinal stenosis Prospective or retrospective studies The articles described the patients who underwent laminectomy or its variant and compared The article compared at least one of the parameters such as operation time/reoperation rate/postoperative complication/adjacent segment diseases The article declared at least of the patient's satisfaction parameters such as JOA, VAS, and ODI scores for the clinical studies.	Articles were published in a language other than English The article was published before 2002 The articles described the fusion procedure Population duplicates from the same institution Spinal stenosis is associated with low- or high-grade spondylolisthesis The patient did not complete the follow-up for clinical evaluation.

### 2.3 Date extraction

Independent reviewers (UD and EM) extracted data from all eligible studies Finally, the articles were grouped by content. All clinical studies were grouped into the following categories, “Operative time,” “Length of hospital stay,” “Postoperative complication,” “Re-operation rate,” “Effect on elderly patients,” “Patient satisfaction,” and “Adjacent segment diseases.” It is obvious that the MIL technique involves less muscle, bone, or ligament removal; hence, the blood loss would be less compared with the laminectomy procedure. Hence, we did not compare the blood losses between these procedures in our review. On the contrary, all biomechanical studies were grouped into the following categories, “FEA study” and “Cadaveric study” where laminectomy was compared with its variant.

## 3 Results

A total of 9 biomechanical studies and 25 clinical studies were selected. In clinical studies, 16 described the mean operative time, 21 stated postoperative complications, 11 reported reoperation rate, 2 reported adjacent segment diseases, 6 reported effects on elderly patients, and almost all studies described patient satisfaction. Numerous studies also described the length of hospital stay for open laminectomy and MIL technique for LSS. Of the biomechanical studies, six FEA studies and three cadaveric studies compared the laminectomy with its variant.

### 3.1 Clinical studies

**Figure 2:**
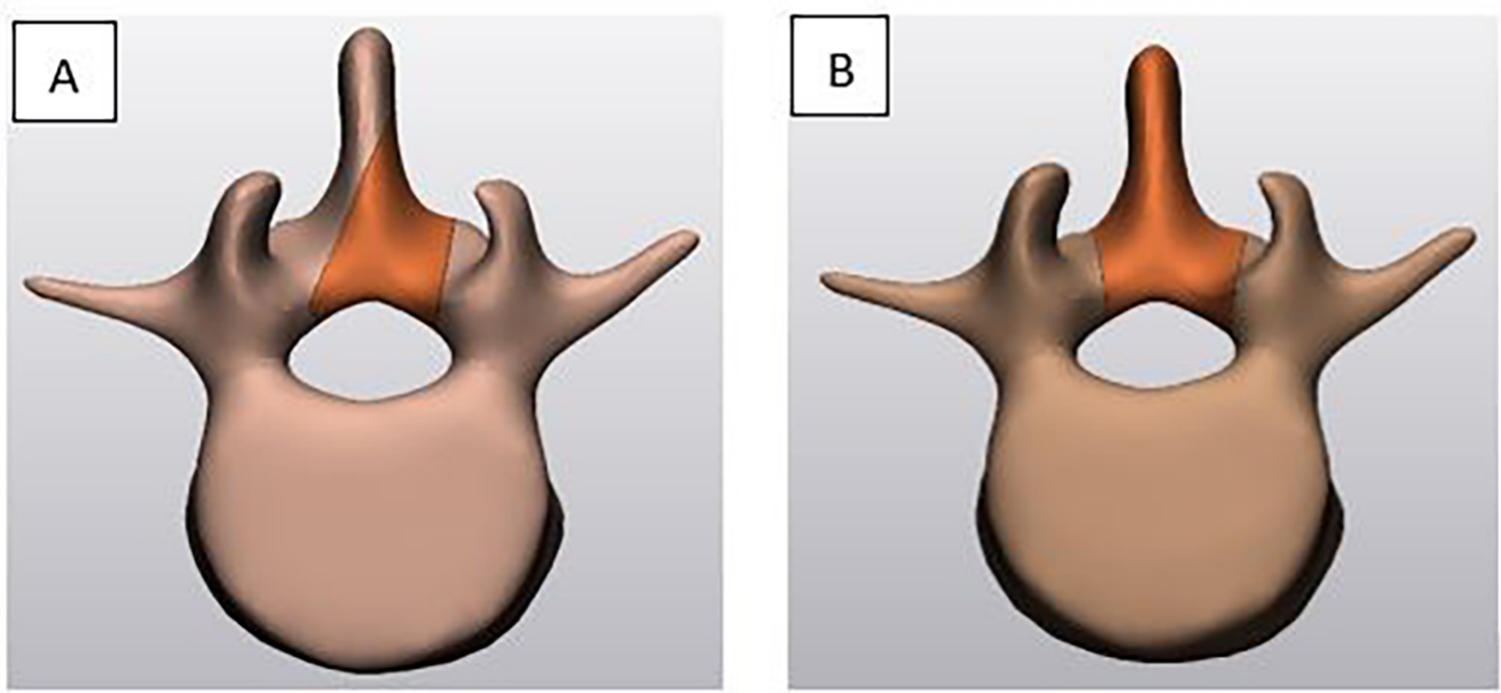
A typical minimally invasive unilateral laminotomy **(A)** versus traditional laminectomy **(B)**.

Clinical studies comparing open laminectomy and MIL techniques for LSS have been conducted to evaluate the outcomes and efficacy of these surgical approaches. We reviewed the various clinical studies regarding operative time, hospital stay, operation level, age, surgical level, postoperative complications, reoperation rate, adjacent segment degeneration, and patients’ satisfaction as it relates to the open laminectomy vs. MIL techniques. Overall, clinical studies suggest that the MIL technique offers advantages such as less hospital stay, lower reoperation rate, fewer postoperative complications, and reduced chance of adjacent segment disease (ASD) than the open laminectomy. Figure 2 shows a typical MIL technique and laminectomy procedure.

#### 3.1.1 Operative time

Most clinical studies mentioned the duration of operation for open laminectomy and MIL technique for LSS. Operative time for surgical procedures can vary depending on various factors, including the complexity of the surgery, the surgeon's experience, patient-specific factors, and the specific technique used. Larger removal of the bone, ligaments, and muscle in the laminectomy leads to longer healing time but shorter operative time.

In this review, the minimum mean operative time was observed from Kanbara et al. (45) and the maximum from Cho et al. (46). In most studies, the MIL technique needs higher operative time than the open laminectomy procedure (28, 46–49). In this review, we found the average operative time per level for the MIL technique was 120 min whereas it was 90 min for the laminectomy. This time frame is the average and may not apply to every individual case.

#### 3.1.2 Length of hospital stay

The length of hospital stay for a laminectomy and MIL for LSS can vary based on several factors such as patient's overall health, the extent of the surgery, and the specific hospital's protocols. Several studies suggested that the MIL technique usually has a shorter hospital stay compared to open laminectomy. Lundberg et al. (20) concluded that for the patients who underwent laminectomy, the average patient's stay in the hospital was 3 days whereas less than 1 day for MIL. Ang et al. (21) also confirmed that the length of hospital stay for open laminectomy was 3 days whereas 1.1 days for the MIL procedure. Multiple clinical studies suggested that there is no significant difference in the length of hospital stay for the number of levels treated in each procedure. For example, Nerland et al. (22) showed two levels and one level laminectomy required an average of 3.1 and 3.4 days for decompression. Similarly, in the MIL group, there was no significant difference for one- or two-level decompression (1.9 days vs. 2.3 days). Mobbs et al. (10) also confirmed that the open laminectomy required a higher length of postoperative hospital stay than the minimally invasive unilateral laminectomy (55.1 vs. 100.8 h).

#### 3.1.3 Postoperative complications

Several studies have examined postoperative complications and factors influencing these complications after surgery for lumbar spine diseases. Both open laminectomy and MIL procedures are safe for lumbar spinal stenosis. Multiple clinical studies demonstrated that elderly patients have a higher postoperative complication rate than that younger patients for both procedures (46, 49–54). These complications include wound infection, cerebrospinal fluid (CSF) leakage, hematoma, and nerve injury in addition to anesthesia-related complications. According to Ikuta et al. (48), the postoperative complication rate in the open laminectomy group was 14% lower compared with the MIL posterior decompression (25%). Additionally, Ang et al. (21) noticed that 3 out of 83 patients had durotomies in the MIL group and no complications for the patients with open laminectomy. Pietrantonio et al. (7) reviewed patients with LSS who underwent open laminectomy or bilateral laminotomy LSS surgical treatment. Clinical outcomes were similar between the two groups, although the complication rate was slightly above for the total laminectomy group than the bilateral laminotomy group. Harrington et al. (55) did not find any difference in postoperative complications for open and MIL procedures. Shih et al. (28) also confirmed that there was no difference in minor postoperative complications between these two procedures (30.1% vs. 34.7%) although only one patient showed wound complication in the open group. Some other researchers such as Rahman et al. (30) found a significant difference in postoperative complications between open laminectomy and minimally invasive decompression (16.1% vs. 7.9%). Details of clinical prospective and retrospective studies are shown in Table 2.

**Table 2:** List of clinical studies for laminectomy and minimally invasive laminectomy techniques.

Author (year)	Study type	Intervention, no. of patient	Mean age in year (SD)	Sex ratio (M/F)	Average operative time (in minutes)	Mean follow-up (in months)	Single/multiple-level	Clinical evaluation
Khoo et al. (2002) (47)	Retro	Laminectomy, 25 MEDL, 25	60.1 68.8	14/11 15/10	88 109	12 12	Multiple Multiple	VAS, SF-36
Ikuta et al. (2005) (48)	Retro	Laminectomy, 29 MEPD, 47	69 66	15/14 23/24	101 124	23 22	Multiple Multiple	VAS, JOA
Thomé et al. (2005) (6)	Pros	Laminectomy, 40 UL, 40 BL, 40	69 ± 10 67 ± 9 70 ± 7	18/22 15/25 20/20	ING	15.5 15.5 15.5	Multiple Multiple Multiple	VAS, SF-36, RMS
Cho et al. (2007) (46)	Pros	Laminectomy, 30 SSPL, 40	58.8 ± 14.9 61.2 ± 10.8	15/15 16/24	193 ± 68 259 ± 122	14.8 15.1	Multiple Multiple	VAS, JOA, PSS
Fu et al. (2008) (56)	Pros	Laminectomy, 76 WT, 76	57.47 ± 5.0 57.78 ± 4.9	33/43 37/39	ING	40 40	Multiple Multiple	VAS, ODI
Harrington et al. (2008) (55)	Retros	Laminectomy, 35 MILM, 31	41.2 42.1	22/13 21/10	84.1 76.8	6 6	Single Single	ODI
Rahman et al. (2008) (30)	Retros	Laminectomy, 88 MID, 38	66.77 ± 1.3 68.89 ± 1.6	ING	157 112	ING	Multiple Multiple	ING
Yagi et al. (2009) (57)	Pros	Laminectomy, 21 UML, 20	70.8 73.3	6/15 8/12	63.6 71.1	18.6 17.8	Single Single	JOA
Erol et al. (2010) (53)	Pros	Laminectomy, 34 MDL, 37	61 ± 13 59 ± 14	16/18 17/20	107 ± 15 83 ± 12	60 60	Multiple Multiple	JOA, ODI
Morgalla et al. (2011) (50)	Retros	Laminectomy, 10 Hemilaminectomy, 45 UPHL, 53	71	5/5 24/21 27/26	ING	ING	Multiple Multiple Multiple	QBPDS, HFBPQ
Shih et al. (2011) (28)	Retros	Laminectomy, 26 MIS, 23	64.5 69.1	12/14 18/5	112.0 141.7	ING	Multiple Multiple	ASA score
Watanabe et al. (2011) (54)	Pros	Laminectomy, 16 LSPSL, 18	71 ± 8 69 ± 10	8/8 10/8	82 ± 36 69 ± 29	12 12	Multiple Multiple	JOA
Ercegovic et al. (2012) (58)	Retros	Laminectomy, 22 MIS, 51	55.8	13/9 24/27	ING	12	Multiple Multiple	RM, VAS
Liu et al. (2013) (49)	Pros	Laminectomy, 29 ULBD, 27	61.1 59.4	18/11 15/12	57 ± 12 67 ± 21	24	Multiple Multiple	JOA, VAS
Parker et al. (2013) (26)	Pros	Laminectomy, 27 MIS, 27	57 ± 11.4	11/16 18/9	ING	24	Multiple Multiple	ODI, VAS
Rajasekaran et al. (2013) (29)	Pros	Laminectomy, 23 LSPSL, 28	54.5 57.3	14/9 16/12	57.1 ± 17.4 62.3 ± 22.1	14.2 ± 2.9	Multiple Multiple	JOA, VAS, NCVAS,NCOS, BPVAS
Ang et al. (2013) (21)	Retros	Laminectomy, 30 MIS, 83	54.7 58.1	10/20 20/54	65 65	24	Single Single	ODI, NASSS, SF-36
Mobbs et al. (2014) (10)	Pros	Laminectomy, 27 ULBD, 27	65.8 ± 14.3 72.7 ± 10.4	ING	ING	44.3 ± 15.0 36.9 ± 4.3	Multiple Multiple	VAS, ODI
Kanbara et al. (2015) (45)	Retros	Laminectomy, 21 LSPSL, 26	69.4 58.3	11/10 18/8	25.7 ± 8.7 22.7 ± 6.2	>12	Multiple Multiple	JOA
Nerland et al. (2015) (22)	Pros	Laminectomy, 414 MIS. 471	70.1 66.6	209/205 249/222	∼102 99	12 12	Multiple Multiple	EQ-5D, ODI
Lundberg et al. (2017) (20)	ING	Laminectomy, 13 MIS, 37	69.7 ± 9.8 64.4 ± 8.3	12/1 36/1	131.25 131.2	ING	Multiple Multiple	Not evaluated
Oichi et al. (2018) (59)	Retros	Lamienctomy, 21781 MEL, 1536	70.4	13,205/8,576 872/664	ING	ING	Multiple Multiple	CCI
Pietrantonio et al. (2019) (7)	Retros	Laminectomy, 105 Bilaterallaminotomy, 109	68.5 70.1	47/58 50/59	ING	120 120	Multiple Multiple	VAS, SF-36. ODI.
Horan et al. (2020) (27)	Pros	Laminectomy, 25 Bilateral laminotomy, 37	63 67	ING	ING	36 36	Multiple Multiple	VAS, ODI
Ovalioglu et al. (2022) (52)	Retros	Laminectomy, SPSD	61.42 ± 11.3 60.54 ± 10.4	52/80 58/86	66.9 ± 18.2 70.4 ± 28.3	38.6 ± 7.2	Multiple Multiple	VAS, ODI

MEDL, microendoscopic decompression laminotomy; MEPD, microendoscopic posterior decompression; SSPL, a split–spinous process laminotomy; WT, windows technique; MILM, minimally invasive lumbar microdiscectomy; MID, minimally invasive decompression; UML, unilateral microendoscopic laminectomy; VAS, visual analog scale; MDL, microdecompressive laminotomy; UPHL, unilateral partial hemilaminectomy; MEL, microendoscopic laminectomy; SPSD, spinous process splitting decompression; SF-36, short-form health survey; JOA, Japanese Orthopaedic Association; RMS, Roland–Morris scale; PSS, Prolo scale score; ODI, Oswestry disability index; Retro, retrospective; Pros, prospective; HFBPQ, Hannover functional back pain questionnaire; QBPDS, Quebec back pain disability scale; ASA, American Society of Anesthesiologists; RM, Roland–Morris; NCOS, neurogenic claudication outcome score; NCVAS, neurogenic claudication VAS; BPVAS, backpain VAS; NASSS, North American Spine Society score; CCI, Charlson comorbidity index; EQ, EuroQol; ING, information not given.

Oichi et al. (59) studied the incidence of postoperative complications between patients treated by microendoscopic laminectomy (MEL) vs. open laminectomy. Analysis of 3,072 individuals—1,536 in each group—revealed that those in the MEL group were significantly less likely to endure a postoperative complication, develop a surgical site infection (SSI), or have postoperative delirium due to longer surgical operation than those in the open laminectomy group. Horan et al. (27) also compared the outcomes of LSS patients treated via two unique surgical approaches—MIL bilateral laminotomy via unilateral approach and open laminectomy. The MIL approach was found to be better among various measures and specifically was shown to have lower complication rates than the open laminectomy.

#### 3.1.4 Reoperation rate

Following lumbar spine surgery, some individuals require reoperation, although the factors putting those patients at higher risk for needing additional surgery are not clearly understood. Several studies have examined postoperative complications and elements influencing reoperation rates after surgery for lumbar spine diseases. Due to inadequate decompression in open laminotomy or MIL procedure, a patient may need revision surgery. Some studies reported that due to CSF leaking, patients need revision surgery in both the laminectomy and MIL groups. Incidental durotomy, scar tissue formation, spinal instability, adjacent segment disease, and recurrent disc herniation are the major reasons for the revision surgery. Out of 25 studies, 11 documented the reoperation rate in this review.

Following open laminectomy or MIL procedure, reoperation risk may be influenced by gender, age, smoking, surgical level, wound infection, hematoma, fusion, etc. Thomé et al. (6) found no significant difference between bilateral laminotomy, unilateral laminotomy, and laminectomy. Because a large amount of bone was removed in the open laminectomy group, postoperative instability was developed, and five patients needed reoperation with instrumentation-assisted fusion. In contrast, no instrumented fusion was required in the bilateral laminotomy group, except for two patients in the unilateral laminotomy group. Mobbs et al. (10) concluded that for the unilateral laminotomy, the reoperation rate was nearly 4%, while it was 11% for the laminectomy for bilateral decompression. According to them, the progression of spinal degeneration may cause a need for reoperation, while wound infection, hematoma, and wound dehiscence cause the rest. In another study, Pietrantonio et al. (7) showed that the open laminectomy group underwent surgery four times more than the laminotomy group. In the laminotomy group, 4 patients required reoperation due to insufficient decompression while 16 patients in the laminectomy group required fusion surgery due to post-laminectomy instability. Horan et al. (27) found that 1 out of 37 patients underwent reoperation following MIL procedures; in contrast, 3 out of 25 following the laminectomy procedure underwent reoperation. In both groups, revision surgery was required due to CSF leakage. As per Ovalioglu et al.’s (52) findings, 5 of 144 patients needed repeated operation after spinous process splitting decompression while 4 of 132 patients for the conventional laminectomy group. According to Erol et al. (53), dural tears occurred in 7 of 34 patients, but none of them, who underwent microdecompressive laminotomy after 5 years of follow-up, needed revision surgery. A prospective study carried out by Morgalla et al. (50) found that 2 out of 108 patients required reoperation and fusion as a result of postoperative instability in the laminectomy, hemilaminectomy, and partial hemilaminectomy groups. Although a CSF fistula was observed in three patients, it was treated using external lumbar drainage instead of revision surgery. Ikuta et al. (48) determined that 2 out of 44 patients needed repeated operation due to the presence of postoperative epidural hematoma in the microendoscopic posterior decompression group whereas 3 out of 29 patients needed revision surgery due to disc herniation in the open laminotomy group. Ang et al. (21) reported that 2 out of 83 patients who underwent the MIL technique required revision surgery due to a higher rate of inadvertent durotomies while in the open laminectomy, no patient needed reoperation at 24 months follow-up. Liu et al. (49) suggested that unilateral laminotomy and conventional laminectomy do not require any revision surgery as no postoperative instability was observed in both groups at the final follow-up. Therefore, most clinical studies suggested that the reoperation rate is not significant between the conventional laminectomy and MIL technique.

#### 3.1.5 Adjacent segment disease

Adjacent segment disease (ASD) occurs when an adjacent segment is adversely impacted following spine surgery, requiring reoperation. When an open laminectomy or MIL is performed on the lumbar spine, the adjacent segment may also be affected. For example, operation on L3–L4 may also impact L2–L3 or L4–L5. The affected segment can be evaluated or determined by clinical evidence or radiological analyses. When comparing open laminectomy and MIL techniques for LSS, the impact on adjacent segment disease is an important consideration. Most of the patients who required reoperation were not for the same level of restenosis. Thomé et al. (6) reported that after unilateral or bilateral laminotomy, there was no adjacent level stenosis except for one patient in a laminectomy procedure. Erol et al. (53) did not find any postoperative adjacent level stenosis in both traditional laminectomy and microdecompressive laminotomy based on 71 patients with 5 years of follow-up.

#### 3.1.6 Effects on elderly patients

For the elderly population, MIL techniques are proposed to be the more suitable procedures for LSS. Khoo et al. (47) concluded that as the microendoscopic decompression laminotomy is associated with less bone and ligament disruption, it is more beneficial to elderly patients offering quicker recovery than an open laminectomy. Additionally, the minimally invasive decompression technique required shorter hospitalization time, and no significant complications were observed during the study. The minimally invasive lumbar spine decompression was found to be both safe and adequate for addressing lumbar degenerative diseases in elderly patients. Erol et al. (53) also suggested that less blood loss and less surgical tissue trauma help the elderly population to early mobilization in the microendoscopic laminotomy procedure. A prospective study carried out by Mobbs et al. (10) suggested that the minimally invasive unilateral laminotomy for bilateral decompression is very beneficial for elderly patients as the postoperative pain and hospital stay are less. In contrast, Yagi et al. (57) stated that the elderly Asian population had relatively smaller vertebrae, making it more difficult for adequate decompression using a unilateral approach. Some researchers such as Nerland et al. (22) did not find a statistically significant difference between laminectomy and microendoscopic decompression procedures for patients aged over 70. Similarly, Morgalla et al. (50) also did not find any difference between laminectomy, unilateral hemilaminectomy, and partial hemilaminectomy by studying patients aged over 60 in 1-year follow-up. The recovery time and improvement of quality of life were similar between the elderly and young patients showed by Aleem et al. (51).

#### 3.1.7 Patient satisfaction

Thomé et al. (6) compared patient satisfaction between 120 LSS patients in one of three treatment groups—those who underwent bilateral laminotomy, unilateral laminotomy, and laminectomy. While there was not a significant difference in lower back pain at rest between groups, more patients in the conventional laminectomy group reported lower back pain during walking than the other two groups. Additionally, the bilateral laminotomy was associated with greater improvements in the visual analog scale (VAS). According to this study, patient satisfaction was significantly higher among patients who underwent the bilateral laminotomy than the other two procedures in treating LSS.

Rosen et al. (1) studied Oswestry disability index (ODI) scores, visual analog scale pain scores, and short-form 36 scores following minimally invasive decompression of lumbar degenerative disease for patients aged 75 years and above. The difference between preoperative and postoperative ODI and VAS scores was significant for the minimally invasive decompression technique. Parker et al. (26) studied 54 patients who underwent one of two treatments—MIL multilevel hemilaminectomy or open multilevel hemilaminectomy—for degenerative LSS. Their primary goal was to analyze the cost-effectiveness of these two procedures. There was no significant difference between each in terms of long-term pain relief, lifestyle, and long-term efficiency. Moreover, they found that the total direct and indirect costs for these two procedures are quite similar (10). In a prospective, 1:1 randomized trial, Mobbs et al. (10) looked at several preoperative and postoperative measures to evaluate minimally invasive unilateral laminectomy for bilateral decompression (ULBD) vs. the standard open laminectomy for LSS treatment. Although there was no significant difference in ODI scores between groups, the difference in VAS scores was significantly better among the ULBD patients compared to those in the open laminectomy group. The patients in the ULBD group also used relatively less opioids than those who underwent open laminectomy. Overall, leg pain, hospital stay, and postoperative pain significantly improved with the ULBD approach compared to the open laminectomy.

### 3.2 Biomechanical studies

Biomechanical studies play a crucial role in comparing different surgical techniques for LSS, such as open laminectomy and MIL techniques. These studies aim to assess the effects of each technique on the biomechanical properties of the spine, including spinal stability, range of motion (ROM), and load distribution. For example, calculating the von Mises stress after the surgical operation in the adjacent intervertebral disc can predict the possibility of adjacent segment degeneration diseases. The changing ROM after open laminectomy or MIL technique can notify how biomechanically stable the spinal column is postsurgery. The larger the removal of posterior elements or ligaments, the greater the risk of spinal stability. We have overviewed six FEA and four cadaveric studies comparing laminectomy and MIL techniques.

#### 3.2.1 FEA studies

Using a motion segment model, Lee et al. (31) evaluated the biomechanical effects of four common decompression approaches—unilateral laminectomy, unilateral laminectomy with unilateral facetectomy, unilateral laminectomy with bilateral facetectomy, and total bilateral laminectomy—for treating LSS. Under all conditions (flexion, extension, bending, torque, anterior or posterior shear), the healthy spine had a higher ROM compared to that of the disc degeneration models. Notably, this research found that for the models in which unilateral or bilateral facet joints were removed, there was a higher ROM compared with the sole laminectomy model. According to the authors, the removal of facet joints increases the annulus stress for all motions. Researchers concluded that total bilateral laminectomy with facetectomy would result in biomechanical instability for most motions.

Ivanov et al. (18) saw similar outcomes for the various MIL techniques through FEA. For younger patients, in most of the MIL models, the ROM did not vary with the intact spine model. However, the ROM was higher when hemilaminectomy was performed. In extension, flexion, and rotation conditions, the highest von Mises stress was observed in the hemilaminectomy model compared with other MIL models. In all physiological loading conditions, the stress on the pars interarticularis was higher in the medial facetectomy and lateral fenestration models. A clinical study suggested (60) that the pedicles are the weakest structure after the pars interarticularis. Hence, increasing stress on the pars interarticularis might lead to stress fracture after such surgeries.

Segmental motion and von Mises stress were studied by Bresnahan et al. (61) in evaluating the effects of graded posterior element removal for treating LSS. They compared two common LSS surgical techniques [complete laminectomy (OPEN) and bilateral interlaminar laminotomies (IL)] with a less invasive approach, the microendoscopic decompression for stenosis (MEDS) procedure, and for each procedure, 25%, 18%, and 25% of the posterior bony elements were removed, respectively. For the flexion condition, the resultant motion of the OPEN model was 1.8-fold higher than the intact model, and in the extension condition, it was four times higher. For left and right axial rotation, the IL or OPEN models had more than two times higher segmental motion than the intact model. There was no difference observed between the three models and the intact model for left and right lateral bending. In all physiological motions, only the MEDS model showed good correlation with the intact model. That means the posterior bony elements can be removed up to 15% without destabilizing effect. The von Mises stress on the annulus was similar in intact, MEDS, and IL models for all loading conditions. In the OPEN model, this stress was 3.6 times higher than the intact model at the surgical level which also led to spinal instability. A summary of biomechanical studies is shown in the Table 3.

**Table 3:** Laminectomy and its variant for biomechanical evaluation for treating LSS.

Author (year)	Study type	Objective	Modeling	Biomechanical conclusion
Lee et al. (2004) (31)	FEA	How laminectomy and facetectomy affect the ROM and biomechanical stability	Four L2–L3 models with different procedures and degenerative model	Bilateral laminectomy with the facetectomy increases the ROM and annulus stresses compared with unilateral laminectomy except for the lateral bending
Ivanov et al. (2007) (18)	FEA	How a MIL technique conducts higher stresses in the remaining neural arch for the young patient with lumbar spinal canal stenosis	Six models with different surgery in the L4 and L5	Higher stress on the pars interarticularis leads to fracture of the pars after the surgery
Bresnahan et al. (2009) (61)	FEA	To analyze the impact on annulus stress and ROM associated with the graded posterior removal	Microendoscopic decompression, interlaminar laminotomy, and open laminectomy model for L1-S1	Laminectomy has the greatest change in ROM for all loading except the lateral bending. Open laminectomy has a higher chance of von Mises stress on the annulus at the surgical level for the flexion condition
Lee et al. (2010) (41)	Cadaveric	To study the laminectomy Vs bilateral laminotomy for the ROM and stiffness	Intact model, bilateral laminotomy, and full laminectomy for L2–L5.	Bilateral laminotomy showed less reduction of stiffness compared with open laminectomy and developed higher stability
Bisschop et al. (2012) (62)	Cadaveric	Stability analysis following laminectomy	Ten cadavers from T12-L5 and laminectomy were performed in L2 and L4	Shear stiffness, shear yield force, and shear force to failure respectively correlated with intervertebral disc degeneration, disc length, and bone mineral density
Bisschop et al. (2014) (38)	Cadaveric	Segmental biomechanical behavior modified after the single-level laminectomy but no influence on the adjacent segments	Twelve cadavers from L1–L5 and laminectomy were performed in L2 and L4	Laminectomy doesn't affect the stiffness but alters the ranges of motion in the adjacent segments for lateral bending
Ahuja et al. (2020) (33)	FEA	Stability analyses by removing unilateral and bilateral facet joint	Nine FEA models with different graded facetectomy	If facet joints were removed by more than 30% during the decompression technique, needed to analyze the spinal stability
Matsumoto et al. (2021) (36)	FEA	Transforaminal full endoscopic lateral recess decompression (TE-LRD) technique for lateral recess stenosis	Intact model, moderate disc and severe disc degeneration model of L4–L5	Although severe disc degeneration could be treated with the 100% TE-LRD, the biomechanical instability showed least at 50% TE-LRD
Lin et al. (2023) (63)	FEA	Evaluating the spinal stability and von Mises stresses on the annulus by removing posterior bone and ligaments	Intact model, unilateral laminotomy, complete laminectomy, and complete laminectomy with facetectomy	Extensive posterior bone and ligaments can increase the ROM in the flexion and axial rotation at the surgical level because facetectomy influenced the load-bearing capacity during flexion

Ahuja et al. (33) carried out a finite element study to determine the effects of removing facet joints in unilateral and bilateral procedures on biomechanical stability. According to this study, the removal of 45% of the unilateral facet joint increased the mediolateral spinal mobility by 30% for both flexion and extension. For the 100% removal of the unilateral facet joint, anteroposterior spinal mobility increased by 40% while mediolateral mobility increased by 80% for flexion. When the facet joints were completely removed on both sides, the anteroposterior mobility increased by 80% in flexion and 90% in extension. In flexion, for the excision of 45% of facet joints, the intradiscal pressure in L4–L5 increased by 30% for unilateral while it increased by nearly 20% for bilateral scenarios. When the facet joint was completely removed, the facet load at L4–L5 increased by more than 100% in extension for bilateral and by 60% for unilateral.

Matsumoto et al. (36) compared the conventional decompression techniques with the graded transforaminal endoscopic lateral recess decompression (TE-LRD) in a moderate disc degeneration model. In normal disc degeneration, although there are differences in 50% and 100% TE-LRD in flexion, the 100% TE-LRD had seven times higher ROM than the 50% TE-LRD in extension. The bilateral laminectomy had a higher ROM than the unilateral for both flexion and extension. The differences between the TE-LRD and all laminectomy models for left and right lateral bending are negligible. In contrast, in the severe disc degeneration model, the difference between 50% and 100% TE-LRD models was negligible for both flexion, extension, and lateral bending loading. Overall, the bilateral laminectomy model had a higher ROM than all models except for rotation. Although 50% TE-LRD increased the facet joint stress, this technique had the least amount of annulus stresses compared with bilateral laminectomy. Hence, 50% TE-LRD was found to be the most effective technique for spinal stability compared with conventional laminectomy or facetectomy. Table 2 shows the FEA and cadaveric studies that involved the open laminectomy and MIL procedures.

Lin et al. (63) investigated how graded posterior bone and ligaments affect the von Mises stresses on the annulus and spinal column by FE analyses. According to their findings, there is little impact on the annulus at the surgical level for the unilateral laminotomy while the ROM increases for the flexion and rotation of the laminectomy associated with the facetectomy. They also created eight layers of the annulus in their models and found that the outmost layer has the maximum von Mises stresses than the inner layer. According to their shear stress analysis, flexion showed higher streses compared with extension, bending, or torque. Overall, when the laminectomy was associated with the facetectomy, the shear stress was approximately 24% higher than the intact model.

#### 3.2.2 Cadaveric studies

Bisschop et al. (38) found that single-level laminectomy does not affect the adjacent segments but increases the ROM at the index level. According to this study, the ROM increased by 7%–12% after single-level laminectomy. For the adjacent level, there were slight effects for lateral bending and no changes were observed for flexion, extension, or axial rotation. It is also interesting to note that the spinal stiffness was not significantly affected in both adjacent segments and index level. Finally, based on the cadaveric biomechanical study, it was concluded that no instrumentation is required for biomechanical stability when the laminectomy is performed at a single level.

To elucidate distinctions between the laminectomy and bilateral laminotomy of the human lumbar spine, Lee et al. (41) used a cadaveric simulation model and measured segmental motion and stiffness. They identified that the average ROM at the surgical level for the bilateral laminotomy increased by 14.3% while this increased by 32% after laminectomy for flexion and extension. After laminotomies, stiffness was reduced by 11.8% and by 27.2% after laminectomy, which was significantly different. Although some have stated there are differences in axial rotation compared to the intact model, Lee et al. (41) did not find any effect for axial rotation or lateral bending. Consequently, the laminotomies resulted in better biomechanical stability than the laminectomy in decompressing the spine.

Another cadaveric study by Bisschop et al. (62) aimed to determine factors influencing spinal stability after lumbar laminectomy. They documented that following the laminectomy procedure the shear stiffness (SS), shear yield force (SYF), and shear force to failure (SFF) declined by 24%, 41%, and 44%, respectively. The SFF had a comparatively lower value in both treated and untreated segments for female specimens. However, bone characteristics were not related to shear stiffness as it was more associated with SYF and SFF. The authors suggest that to evaluate postoperative instability risk, it is important to know the patient's disc shape, bone mineral contents, or density rather than the pedicle or facet geometry, as pedicle sections and facet geometry did not effectively forecast instability after lumbar laminectomy.

Bisschop et al. (37) also investigated how shear loading affects the biomechanical stability of the human lumbar spine after laminectomy. The authors present that bone mineral density (BMD) and disc degeneration affect torsional strength and stiffness respectively. [In both untreated and laminectomy groups, the early torsion stiffness (ETS) or late torsional stiffness (LTS) is increased with the BMD.] Similarly, ETS or LTS also increases with degeneration for both untreated and laminectomy groups. According to their study after laminectomy, there was an approximately 44% reduction in the strength for shear loading while the reduction in stiffness was about 20%.

## 4 Discussion

LSS is a medical condition that occurs when the spinal canal in the lumbar spine narrows, putting pressure on the spinal cord and nerves. One of the challenges is that the effectiveness of different treatments can vary widely depending on the individual patient.

Most articles showed the average operating time for the MIL technique to be higher than the laminectomy procedure. In our review, only a few studies did not find the differences between the two techniques (20, 21). The length of hospital stays for an open laminectomy and MIL technique can vary depending on several factors, including the patient's specific condition, the complexity of the surgery, and the surgeon's approach. Most of the clinical studies showed that the traditional laminectomy typically requires a longer hospital stay than the MIL technique. According to our review, the average hospital stay for the laminectomy procedure and MIL technique was 3 days and 1 day, respectively (21). The patients who underwent one- or two-level open laminectomy or MIL procedure for LSS required the same length of hospital stay (22). Because of the lengthier hospital stay, clinical studies show that the hospitalization cost is also higher in the open laminectomy procedure than in the MIL technique (20).

When comparing the complication rates for open laminectomy and MIL techniques in the treatment of LSS, it's essential to consider that specific complications can vary depending on various factors such as patient characteristics, surgeon expertise, and the specific procedure performed. Some researchers suggested (7, 21, 22, 45, 58) that the postoperative complication rate is higher in the total laminectomy than the unilateral or bilateral laminectomy. Because of larger soft tissue disruption, the surgical site infection could be higher in open laminectomy (59). However, some studies did not find any significant difference between the two procedures when comparing the postoperative complication rate (27, 28).

Regarding comparing reoperation rates for open laminectomy vs. MIL techniques for LSS, it is important to note that specific rates can vary depending on the study, surgeon's experience, patient characteristics, and other factors. Unintentional durotomy, inadequate decompression, postoperative instability, scar tissue development, spinal instability, adjacent segment degeneration, and recurrent disc herniation are the primary sources for revision surgery. The majority of studies (6, 7, 10, 27, 53, 56) have indicated that MIL techniques result in lower postoperative complication rates, which in turn lead to reduced reoperation rates compared to traditional laminectomy. In contrast, only one clinical study (21) manifested that the MIL technique required more revision surgery than the conventional laminectomy due to inadequate decompression. Some studies (49, 52) did not find significant differences between these two procedures when compared to the reoperation rate for the LSS. Overall, the literature suggested that the reoperation rate for laminectomy ranged from 7% to 20%, while the reoperation rate for MIL techniques ranged from 2% to 10%.

In this review, only two papers reported the adjacent segment disease after open laminectomy or MIL procedure. Of them, only one patient experienced adjacent segment disease after an open laminectomy procedure. Older patients have been bringing osteophytes that reduce mobility; in these patients, stability may not be affected after MIL or open laminectomy procedure. Many papers (10, 47, 53) suggested that less bone disruption and less postoperative pain are more beneficial to the elderly population as it allows early mobilization. A small number of studies (22, 50) did not find significant difference between these procedures when comparing the young and elderly patients.

Patient satisfaction with the surgical treatment of LSS can depend on several factors, including the specific procedure performed, individual patient experiences, and expectations. Many articles (1, 6, 10) suggested that the VAS, ODI, and Japanese Orthopaedic Association (JOA) scores improved in the MIL group compared to open laminectomy. Some studies (16, 64, 65) have suggested that open laminectomy may be associated with a higher risk of the adjacent segment disease. The removal of the lamina alters the stability and load distribution of the spine, potentially leading to increased stress on the adjacent segments over time. This increased stress can contribute to the degeneration of the adjacent discs or the development of spinal instability.

Biomechanical studies showed that the von Mises stress on the upper adjacent level was relatively higher than the lower adjacent level. Furthermore, it has been clinically shown that the rostral side has higher ASD than the caudal side when patients were treated by laminectomy, MIL, or lumbar fusion procedure (64). In biomechanical studies (34, 38, 66), we observed that measuring the stress on the annulus fibrosus is of greater interest, as the stress changes in the nucleus are not significant. In the cadaveric study, one researcher (41) suggested that when the laminectomy involved only a single level, no instrumentation was required as the single-level laminectomy did not significantly affect the total spinal biomechanics column.

## 5 Conclusions

LSS is a complex condition that requires careful diagnosis and individualized treatment. With ongoing research and innovation in this field, we will likely continue to see improvements in diagnostic techniques, treatment options, and outcomes for patients with this condition. This study suggests that MIL may have a lower reoperation and complication rate than open or traditional laminectomy. As the MIL involves less bone disruption, this technique may be biomechanically more stable. Despite these challenges, advances in medical research have led to improved diagnostic and treatment options for LSS. In addition, studies have shown that exercise programs and other conservative measures can be effective in managing symptoms and improving the quality of life for patients with LSS. Minimally invasive spine surgeries have gained popularity in recent years due to their potential benefits, including smaller incisions, less tissue disruption, reduced postoperative pain, and quicker recovery times. These advantages often contribute to shorter hospital stays compared to traditional open procedures.
